# Supplementary material for: The Xpert® MTB/RIF diagnostic test for pulmonary and extrapulmonary tuberculosis in immunocompetent and immunocompromised patients: Benefits and experiences over 2 years in different clinical contexts
Source: PLoS One. 2021 Mar 3;16(3):e0247185. doi: 10.1371/journal.pone.0247185 (PMC7928506; doi:10.1371/journal.pone.0247185)
Supplement: Attachment — Submitted filename: Reviewers comments_181020.docx [file pone.0247185.s001.docx]

**Title:** **Xpert® MTB/RIF diagnostic test for pulmonary and extrapulmonary tuberculosis in immunocompetent and immunocompromised patients: benefits and experiences over 2 years in different contexts**

**Summary:** This prospective observational study aimed to evaluate the performance of Xpert MTB/RIF in different contexts as listed below

(i) laboratory and clinical/epidemiological diagnosis;
(ii) HIV-positive and -negative populations; and
(iii) type of specimens: pulmonary and extrapulmonary.

The Xpert MTB/RIF results were compared with those of Ziehl–Neelsen stained smears and BAAR cultures on solid L.J medium for specimens belonging to clinically suspected TB cases during a two-year period. Total, 924 specimens (pulmonary 514/55.7% and extrapulmonary 410/44.3%) from 743 patients were evaluated. Among these, according to clinical criteria, 101 (13.6%) were classified as confirmed TB cases, 36 (4.8%) probable TB and 606 (81.6%) improbable TB cases. Overall sensitivity, specificity, positive predictive value, and negative predictive value results of Xpert MTB/RIF compared to those of the L.J cultures were 96%, 94%, 57%, and 99%, respectively. The Xpert MTB/RIF test showed an increase in the detection of 13

(35.1%) of 37 confirmed cases of TB in patients with advanced immunosuppression showing negative culture.

**Recommendation:** Accept with major revisions as described below

**Comments:**

1. Phenotypic culture confirmatory method used in this study (L.J culture) is conventional and very suboptimal reference standard for detection of extra-pulmonary and paucibacillary TB. In HIV-positive patients, the diagnosis of pulmonary tuberculosis (PTB) is a challenging task due to the paucibacillary nature of the infection, which greatly reduces the effectiveness of smear microscopy and conventional L.J. culture techniques that are used as a phenotypic reference standard to confirm TB in this study. So, I would recommend authors to use composite reference standard (CRS) as a gold standard to evaluate the true diagnostic potential of Xpert MTB/RIF assay and other diagnostic tests for detection extra-pulmonary and paucibacillary TB. Calculation of sensitivity, specificity, PPV and NPV of all laboratory tests against CRS+ and CRS - patients would give more realistic picture.
2. Clarity in writing and flow need to be improved especially in abstract, methods, results and discussion sections.
3. **Abstract:** Xpert® MTB/RIF assay was first approved for diagnosis of pulmonary tuberculosis and RIF resistance by WHO in 2010. Revised guidelines were published by WHO in 2013 with recommendations for diagnosis of pediatric and extra-pulmonary TB especially CSF, tissue and lymph-node samples. Authors should make these corrections.
4. **Material & Methods:**
(a) **Study design, patient population, and samples, Lines 77-79**

One of the exclusion criteria was patient’s samples that did not yield AFB culture and/or smear results. Authors should elaborate on this with reasons as the traditional phenotypic methods (AFB smear and L.J culture) used in this study itself have many limitations especially while detecting paucibacillary TB from extrapulmonary samples &/or samples collected from HIV patients etc. Further, it would be interesting to know issues related to unavailability of AFB smears results.

(b) **Study design, patient population, and samples, Line 100**

Symptomatic patients without any established diagnosis are classified as improbable TB group. Means all the patients included in this group have clinical symptoms and/or signs suggestive of TB but radiology, ZN smear, AFB culture and other tests results are negative for TB but it. Further, phenotypic & other conventional reference methods (AFB smear and/or L.J culture, radiology, histopathology/cytology, etc.) utilized this study have known limitations in detection of TB especially paucibacillary TB from extrapulmonary specimens. Therefore, considering limitations of technology used as a reference standard, possibility of TB disease cannot be eliminated in this group of symptomatic patients. I would recommend changing the terminology improbable TB group as “possible TB”.

**(c)** **Laboratory Methods: Line 109-111**

Xpert MTB/RIF assay protocol for sample processing for urine to be described

as it is not recommended by manufacturers as well as WHO.

5. **Results:**
(a) **Study population: Lines 129 -130**

Authors mentioned that, of the total, 743 patients included in the study; 616 patients underwent BAAR (smear), culture, and Xpert MTB/RIF tests with a single sample, whereas 310 patients underwent these tests using multiple samples. If we add these two groups of patients (616 with single sample and 310 with multiple samples) then total number of patients are 926 not 743. Kindly clarify number of patients and total number of samples.

(b) **Study population: Lines 131 to 142**

For better understanding, all the patient’s details can be incorporated in a table format. Flow chart with total number of patients & samples, clear data on number of samples excluded, among remaining samples how many collected from of HIV positive and HIV negative patients, number of pulmonary and extra-pulmonary specimens etc. to be included.

(c) **Comparison against composite reference standards (CRF):** In result section, table comparing results of ZN smear, L.J culture, Xpert MTB/RIF assay against composite reference standards (CRS) to be included. Sensitivity, specificity, PPV & NPV of these tests against CRS can be included in the same table.
6. **Discussion:** Discussion should be more precise and to the point. Discussion on Xpert ultra (lines 291 -305) is unnecessary. Authors should discuss their results in detailed and compare it with other relevant published data.
(a) **Comparison of Xpert CT values with AFB smear: Line 258-260**

Table showing comparison of Xpert CT values with ZN smear gradation among different patient population (pulmonary & extra-pulmonary, HIV+ & HIV- etc.) can be incorporated. Further, Xpert CT values can be comparted against L.J culture turnaround time in days among different patient population.

(b) In this study, there were only 7 RIF resistant cases. Of these three are form same patients. So only 7 samples collected from 5 patient won’t be enough number of samples to discuss on performance of Xpert MTB/RIF assay for detection of RIF resistance.
(c) **False RIF resistance calls (n=4): line 273-274**

Few low-level RIF resistant mutants are phenotypically susceptible when DST is performed using liquid culture systems like BACTEC 460 TB and MGIT 960 TB system. So further confirmation of four Xpert resistant, phenotypically susceptible TB strains by other molecular methods like Line Probe Assay or sequencing is recommended for confirmation.

7. **References:** All references should be written properly as per PLOSOne guidelines. Journal name & proper page numbers missing in few references. Please recheck and make necessary corrections.
